# Sensitivity Enhancement of Two-Dimensional Materials Based on Genetic Optimization in Surface Plasmon Resonance

**DOI:** 10.3390/s19051198

**Published:** 2019-03-08

**Authors:** Guo Xia, Cuixia Zhou, Shiqun Jin, Chan Huang, Jinyu Xing, Zhijian Liu

**Affiliations:** 1 National Engineering Laboratory of Special Display Technology, National Key Laboratory of Advanced Display Technology, Academy of Photoelectric Technology, Hefei University of Technology, Hefei 230009, China; zhcc@mail.hfut.edu.cn (C.Z.); shq_king@163.com (S.J.); 2 Anhui Institute of Optics and Fine Mechanics, Chinese Academy of Sciences, Hefei 230031, China; choptics@mail.ustc.edu.cn; 3 University of Science and Technology of China, Hefei 230026, China; 4 Key Laboratory of Optical Calibration and Characterization, Chinese Academy of Sciences, Hefei 230031, China; 5 School of Instrument Science and Opto-Electronics Engineering, Hefei University of Technology, Hefei 230009, China; xingjinyu@mail.hfut.edu.cn (J.X.); 13721021965@163.com (Z.L.)

**Keywords:** surface plasmon resonance, sensitivity, two-dimensional materials, sensor

## Abstract

Sensitivity is an important performance index for evaluating surface plasmon resonance (SPR) biosensors. Sensitivity enhancement has always been a hot topic. It is found that the different refractive indices of samples require different combinations of prism and metal film for better sensitivity. Furthermore, the sensitivity can be enhanced by coating two-dimensional (2D) materials with appropriate layers on the metal film. At this time, it is necessary to choose the best film configuration to enhance sensitivity. With the emergence of more and more 2D materials, selecting the best configuration manually is becoming more complicated. Compared with the traditional manual method of selecting materials and layers, this paper proposes an optimization method based on a genetic algorithm to quickly and effectively find the optimal film configuration that enhances sensitivity. By using this method, not only can the optimal number of layers of 2D materials be determined quickly, but also the optimal configuration can be conveniently found when many materials are available. The maximum sensitivity can reach 400°/RIU after optimization. The method provided application value for the relevant researchers seeking to enhance sensitivity.

## 1. Introduction

Surface plasmon resonance (SPR) was developed in the 1990s to detect the interaction between ligands and analytes on biosensor chips [1]. SPR is the resonant oscillation of conduction electrons at the interface between noble metal and dielectric stimulated by incident light [2]. In recent years, SPR sensors have been rapidly developed and applied in many fields, such as drug selection [3], clinical diagnosis [4], food detection [5], environmental monitoring [6], forensic identification [7], and have also become a standard biophysical tool [8]. These developments have occured due to the advantages that SPR sensors possess, including no labeling, fast analysis speed, strong specificity and dynamic measurement.

Surface plasmon resonance (SPR) sensors are valuable tools to study the interaction and sensing of gases and biomaterials, and sensitivity is an important parameter to evaluate its performance. In the last two decades, many methods were proposed to improve the sensitivity of SPR sensors. Zynio et al. reported a method for the enhancement of sensor sensitivity using bimetallic layers [9]; Gupta et al. selected a coupling prism according to the work requirements to improve the sensitivity [10]; Kim at al. enhanced sensitivity in SPR biosensors by the use of periodic metallic nanowires [11]. With the advent of 2D materials, their unique electrical and optical properties have attracted extensive attention. Their applications in sensing technology have also attracted wide attention [12]. Li at al. enhanced sensitivity by plating graphene on gold film [13]; Verma at al. increased the sensitivity of SPR based on biomolecules sensor using graphene and silicon layers [14]; Ouyang at al. reported a method for the enhancement of the sensitivity with transition metal dichalcogenides (TMDCs)/silicon nanostructure plating on gold film [15]; Maurya et al. improved the performance of SPR biosensors based on graphene or MoS_2_ using silicon [16]; Wu at al. proposed a structure of Ag + black phosphorus(BP) + graphene/ TMDCs to improve SPR sensitivity [17]; Meshginqalam at al. put forward a structure of Au + BP + graphene/TMDCs to improve sensitivity of SPR biosensors for sensing DNA hybridization [18]. Xu et al. proposed a TMDCs-metal structure with high imaging sensitivity and detection accuracy [19,20]. Xu et al. also proposed a method that involves combining Ti_3_C_2_T_x_ MXene and TMDCs to enhance the sensitivity [21]. These methods to enhance sensitivity by changing the coupling prism or metal film or adding new materials to the metal film need to select the best combination under a variety of combinations. Using manual debugging options can be time-consuming and may not necessarily find the best configuration.

The thickness of each layer in the SPR sensor structure will affect its sensitivity. Choosing the best thickness will also increase its sensitivity. Genetic optimization algorithms can be used to select the best thickness. Bahrami at al. proposed an improved refractive index (RI) sensor based on genetic optimization of plasmon waveguide resonance to enhance the sensitivity [22]; Pellegrini at al. designed a high-performance magnetooptic surface plasmon resonance (MOSPR) sensors using a multi-objective optimization approach [23,24]; Benazize at al. enhanced sensitivity by optimizing the incidence angle and metal film thickness of SPR-sensor based on graphene-silver substrate [25]. When the structure is fixed, the sensitivity of the structure can be improved by using genetic algorithm to optimize the thickness of the membrane. But it is not necessarily possible to obtain the best sensitivity in the refractive index range of the sample. In this paper, a genetic algorithm is used to enhance the sensitivity of the multi-layer structure. The thickness of metal film and the number of layers of 2D materials (BP/graphene/MoS_2_/WS_2_/MoSe_2_/WSe_2_) are put into the optimization algorithm as the optimization parameters. Then the optimal configuration is obtained to determine the thickness of metal film, the type of 2D materials and the number of layers of 2D materials.

## 2. Modeling

**Figure 1:**
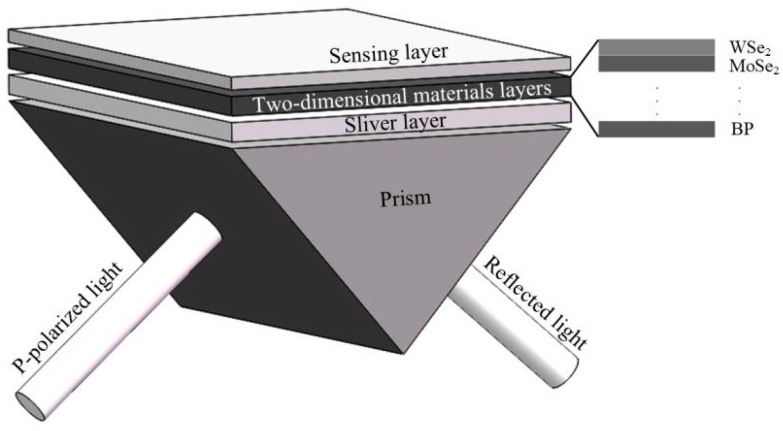
Diagram of an SPR sensor.

In the article an N-layer angular SPR biochemical sensor in the Kretschmann configuration is proposed by using heterostructures of a few-layer 2D materials to enhance the sensitivity. The specific structure is shown in Figure 1. In the structure, we use BK7 glass as the coupling prism and a certain thickness of silver (Ag) film as the noble metal for exciting SPP. Then the silver film is coated with two dimensional materials. The refractive index of BK7 can be calculated through the following dispersion relation

(1) $$n_{BK7}=\left(\frac{1.03961212\lambda^{2}}{\lambda^{2}-0.00600069867}+\frac{0.231792344\lambda^{2}}{\lambda^{2}-0.0200179144}+\frac{1.03961212\lambda^{2}}{\lambda^{2}-103.560653}\right){}^{1/2},$$

The refractive indexes of metals can be calculated through the following Drude–Lorentz model

(2) $$n_{m}=\sqrt{\varepsilon_{m}}={\left[1-\frac{\lambda_{c}\lambda^{2}}{\lambda_{p}{}^{2}(\lambda_{c}+i\lambda )}\right]}^{1/2},$$

where $\lambda_{p}$ (1.4541 × 10^−7^ m) and $\lambda_{c}$ (1.7614 × 10^−5^ m) represents the plasma and the collision wavelengths of silver, respectively. The working wavelength chosen in this paper is 633 nm. Table 1 is the refractive index of two dimensional materials and its corresponding layer thickness of some materials, including black phosphorus (BP), graphene and TMDCs (MoS_2_, WS_2_, MoSe_2_, WSe_2_) [15,16,17,18].

In order to further describe the relationship between the reflectivity of SPR sensor and each film layer, the transfer matrix method is employed. The N-layer structure where $n_{k}$ is the complex values of the RI and *k* is the permittivity of the *k*th layer with thickness $d_{k}$. The characteristic matrix of the N-layer structure can be expressed by

(3) $$\begin{array}{ll} M & =\prod_{k=2}^{N-1}M_{k}=\left[\begin{array}{cc} M_{11} & M_{12}\\ M_{21} & M_{22} \end{array}\right]\\ & =\left[\begin{array}{cc} \cos\beta_{k} & -i\sin\beta_{k}/q_{k}\\ -iq_{k}\sin\beta_{k} & \cos\beta_{k} \end{array}\right] \end{array}$$

(4) $$\beta_{k}=(2\pi d_{k}/\lambda )(\varepsilon_{k}-n_{1}^{2}\sin^{2}\theta_{1}{)}^{1/2},$$

(5) $$q_{k}={\left(\varepsilon_{k}-n_{1}^{2}\sin^{2}\theta_{1}\right)}^{1/2}/\varepsilon_{k},$$

The reflection coefficient $r_{p}$ of the p-polarized (TM-polarized) incident wave can be expressed as

(6) $$r_{p}=\frac{\left(M_{11}+M_{12}q_{N})q_{1}-(M_{21}+M_{22}q_{N}\right)}{\left(M_{11}+M_{12}q_{N})q_{1}+(M_{21}+M_{22}q_{N}\right)},$$

and therefore, the reflectance $R_{p}$ is $R_{p}={\left|r_{p}\right|}^{2}$.

Sensitivity is one of the important parameters of the SPR sensor. The RI sensitivity of the SPR sensor can be defined as the ratio of the change in value of the resonance angle to the change in value of refractive index of the analyte, when the RI of the analyte changes slightly. The formula can be expressed as

(7) $$S=\frac{\delta \theta_{res}}{\delta n},$$

where *S* stands for sensitivity, $\delta \theta_{res}$ stands for resonance angle offset, and δn represents the refractive index change in value of the sample to be detected.

In order to better evaluate the performance of SPR sensors, a figure of merit (*FoM*) is defined. This *FoM* is the product of the sensor’s sensitivity (S) and the full width at half maximum (*FWHM*). The formula can be expressed as [18]

(8) $$FoM=\frac{S}{FWHM}$$

## 3. Genetic Optimization

Genetic Algorithm (GA) [26,27] is a computational model simulating the natural selection and genetic mechanism of Darwinian biological evolution. It is a method used to search for the optimal solution by simulating the natural evolution process. GA starts with a population that represents the potential solution set of the problem. After the emergence of the first generation of population, according to the principle of survival of the fittest, more good approximate solutions have evolved from generation to generation. In each generation, individuals are first selected according to the fitness of the individuals in the problem domain, and then crossover and mutation are carried out by means of genetic operators of natural genetics to produce populations representing new solutions.

**Figure 2:**
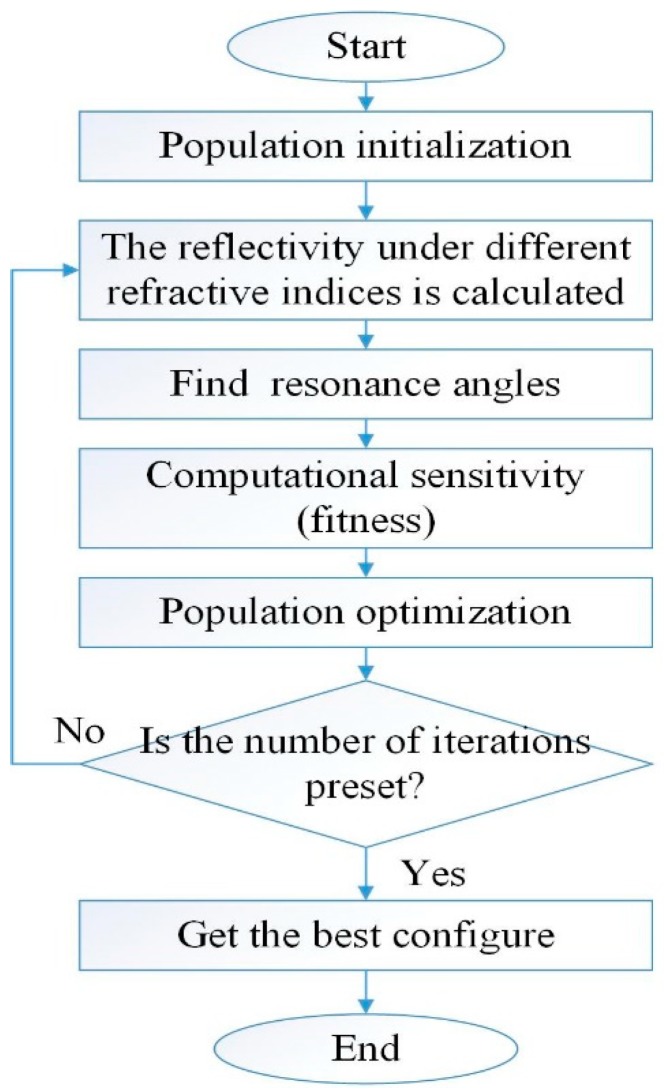
General flow chart of the overall operation.

Sensitivity is selected as a fitness function. The thickness of silver film and the number of layers of 2D materials are the coding factors of population. The specific flowchart is shown in Figure 2.

The first step is to randomly select 100 sets of data as the initial population according to the predefined approximate range (silver film thickness 1–100, the number of layers of each material 0–20). The second step is to calculate the reflectivity of the samples with different refractive indices according to the initialized population. The third step is to find the resonance angle according to the reflectivity curve and calculate the sensitivity. The fourth step is population optimization based on genetic operators. Steps 2, 3, 4 are executed in loop until the number of iterations is satisfied. Finally, the best ratio was obtained.

## 4. Results and Discussion

Due to the unique electrical and optical properties of 2D materials, their applications in biosensors have also attracted widespread attention. It has been pointed out in some articles that different kinds of 2D materials or different layers of the same material on the metal layer can cause varying degrees of sensitivity changes. In this paper, the best configuration of multilayer structure is obtained through genetic optimization. This optimal configuration may be a single 2D material or a combination of various 2D materials. The feasibility of this method is discussed through specific examples and their results.

In order to verify the feasibility of the method, two kinds of SPR structures are optimized and verified. First, according to the reference verification and analysis [17], the results are as follows.

Genetic optimization is carried out according to reference [17]. The whole can be divided into five optimization situations: Silver -BP- graphene/MoS_2_/WS_2_/MoSe_2_/WSe_2_. The silver and BP layers are fixed in references, but silver and BP are also taken into account in genetic optimization. The sensitivity of references and optimization method is listed in Table 2 and Table 3.

In order to further verify the feasibility, genetic optimization is carried out in accordance with reference [18]. The optimization results are shown in Table 4.

Comparing the sensitivity of the proposed method with the sensitivity of the literature, it can be seen that this method is feasible to enhance sensitivity by using GA. The maximum sensitivity of some refractive indices can be enhanced by optimization, and the average maximum sensitivity of some refractive indices can also be increased. This section is based on the determination of 2D materials needed to optimize the number of layers of material and get a higher sensitivity configuration. In the next section, the selection of 2D materials and the number of layers of each material are optimized as variables to select the best configuration.

According to the content of the previous section, it is feasible to optimize the multi-layer structure by genetic algorithm to get the best film configuration with the best sensitivity in the range of the refractive index. Now the optimized structure shown in Figure 1 is selected. The part to be optimized is the thickness of the metal layer and the number of layers of BP/graphene/MoS_2_/WS_2_/MoSe_2_/WSe_2_. The refractive index range of the sample is 1.33–1.355, and the variation interval is 0.005. In this structure, a variety of 2D materials are coated on the metal film. Then an optimal film configuration is obtained by the optimizing method in a certain refractive index range. By using this method, the optimal configuration can be selected with high sensitivity, and then the required experimental materials can be determined conveniently and quickly. The optimization results are shown in Table 5.

**Figure 3:**
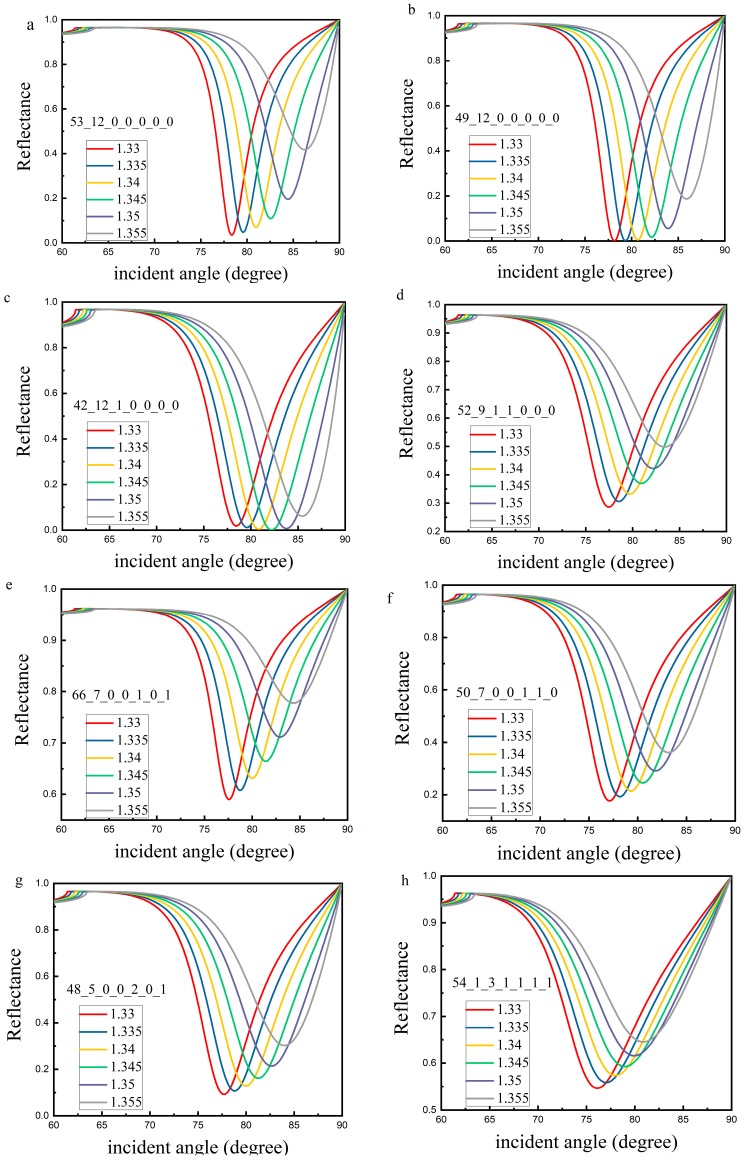
SPR curves changes when the refractive index of sensing medium changes from 1.33 to 1.355 under the set of parameter values in Table 5. (**a**–**h**) corresponds to the configuration 1–8 in Table 5, respectively.

**Figure 4:**
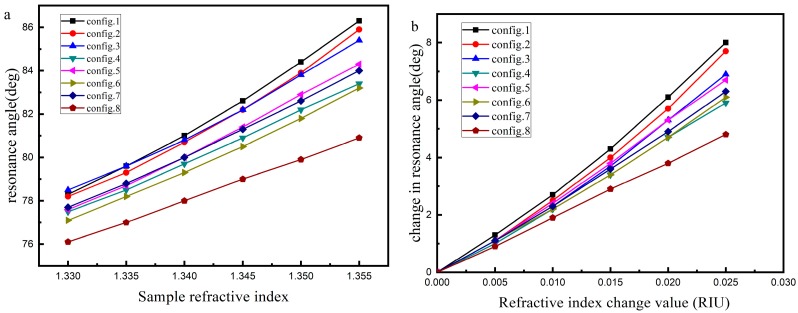
(**a**) The resonance angle and (**b**) the variation of resonance angle obtained by changing the refractive index of analyte.

**Figure 5:**
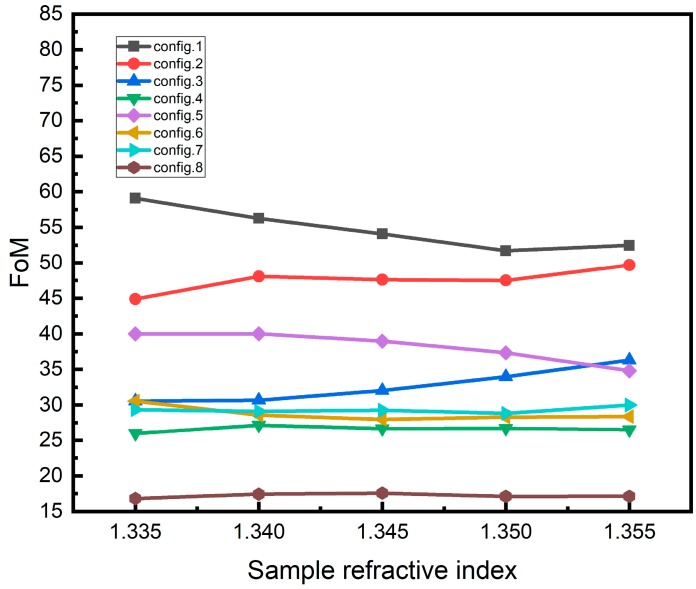
The figure of merit obtained by changing the refractive index of analyte.

Because the size of population is 100 and the optimal configuration of each generation can be optimized, the optimal configuration of optimization can be more than one group. Table 5 lists several representative configurations. According to the parameters set in Table 5, the reflectance curves and sensitivity with different sample refractive index can be obtained. The results of reflectance curves are shown in Figure 3. The resonance angle and the variation of resonance angle obtained by changing the RI of analyte is shown in Figure 4. Figure 4a shows the resonance angle corresponding to the RI of the sample. Figure 4b shows a change in the resonance angle corresponding to the change in RI, and the RI starts from 1.330. Figure 5 shows the FoM of the corresponding RI with the RI ranges from 1.330 to 1.355.

The optimal configuration of each optimization may be more than one set. The data in Table 5 show that the maximum RI sensitivity of a certain refractive index may be obtained at a certain parameter configuration, but the average sensitivity in the refractive index range of all samples may not be the maximum. Similarly, it is possible to obtain the maximum average sensitivity in the refractive index range of all samples at a certain parameter configuration, but the maximum RI sensitivity is not necessarily the maximum. For the first two sets of data in Table 5, the maximum RI sensitivity is 380°/RIU (RI: 1.350–1.355) and the average RI sensitivity is 320°/RIU in the first group. The maximum RI sensitivity is 400°/RIU (RI: 1.350–1.355) and the average RI sensitivity is 308°/RIU in the second group. It can be seen that the most sensitive parameter configuration does not necessarily yield the best average. The results of Table 3 and Table 5 show that the optimum configurations may vary when the refractive index ranges of the samples are different, even if the initial values of the refractive index of the samples are the same.

The results of Table 5, Figure 4 and Figure 5 show that the FoM corresponding to high sensitivity is not necessarily high due to the FWHM. In the RI range, the sensitivity obtained by fitting according to Figure 4 is close to linear function and quadratic function. The results of the linear function are 320°/RIU, 307.43°/RIU, 277.14°/RIU, 238.86°/RIU, 271.43°/RIU, 242.86°/RIU, 252.57°/RIU and 192.57°/RIU respectively, which are close to the average. It can be found from the sensitivity and FoM that in the all optimal configuration, silver + BP or silver + BP + one or two of the 2D materials (graphene/ MoS_2_/WS_2_/MoSe_2_/WSe_2_) have better sensitivity. The sensitivity of silver + BP + graphene+ MoS_2_+WS_2_+MoSe_2_+WSe_2_ is rather poor. In actual use, the best configuration can be chosen according to the actual situation.

## 5. Conclusions

In this paper, an optimization method is proposed to quickly select the best sensitivity configuration. Taking the multi-structure of angle modulation as an example, the optimal thickness of metal film and the number of layers of 2D materials can be selected from a variety of 2D materials to enhance the sensitivity of the sensors.

The first part of Chapter 4 shows that sensor sensitivity can be enhanced by optimizing the number of layers of 2D material when the structure and materials are determined. The following part shows that when the structure is uncertain, the multi-layer structure can be optimized simultaneously to select the optimum film configuration and to enhance the sensitivity. In this way, the best configuration of existing materials can be selected through optimization, and the materials can be selected according to the best configuration. Table 3 and Table 4 show the optimization results for determining 2D materials and SPR structure. In Table 5, a number of optimal configuration representatives are selected when the structure is uncertain. The RI sensitivity can be increased to 400°/RIU when the refractive index of the sample is in the range of 1.350–1.355 and the parameter configuration is 49 nm thick silver film and 12 layers of BP. The maximum average can reach 320°/RIU when the parameter configuration is 53 nm thick silver film and 12 layers of BP. Because of the influence of FWHM, the FoM may decrease with the increase of sensitivity, which depends on the growth rate of both. When the growth rate of sensitivity is greater than FWHM, the FoM increases as the sensitivity is enhanced. In contrast, while the sensitivity is enhanced, the smaller FoM decreases.

The optimization results show that the sensitivity is not always desirable when there are many kinds of added 2D materials. When a variety of materials of the same type can be selected, the optimal combination can be quickly and effectively selected according to the optimization method. Although only one structure has been put forward in the paper, this method is applicable not only to this structure but also to other modulations and materials SPR sensors. It has provided reference value for assisting relevant researchers to select the best configuration effectively and conveniently.

## Figures and Tables

**Table 1:** The refractive index and thickness of different types of 2D materials at λ = 633 nm.

Type of 2D Materials	Thickness of Monolayer (nm)	Refractive Index
BP	0.53	3.5 + 0.01i
graphene	0.34	3 + 1.1491i
MoS_2_	0.65	5.08 + 1.1723i
WS_2_	0.8	4.9 + 0.3124i
MoSe_2_	0.7	4.62 + 1.0063i
WSe_2_	0.7	4.55 + 0.4332i

**Table 2:** Original results in reference [17].

Type of 2D Materials	Layers (L)	Sensitivity (°/RIU)
graphene	5	217
MoS_2_	1	218
WS_2_	1	237
MoSe_2_	2	229
WSe_2_	2	279

**Table 3:** Genetic optimization.

Sensor Structure	Ag (nm)	N	L	S (°/RIU)
Ag +N*BP+L*Graphene	65	12	2	300
Ag +N*BP+L*MoS_2_	55	11	1	280
Ag +N*BP+L*WS_2_	56	11	1	340
Ag +N*BP+L*MoSe_2_	47	12	1	280
Ag +N*BP+L*WSe_2_	50	12	1	340

**Table 4:** Genetic optimization.

Sensor Structure	Au (nm)	N	L	S_max_ (°/RIU)	Save (°/RIU)
Au	55	0	0	90	78
Au +N*BP	49	13	0	190	144
Au +N*BP+L*Graphene	50	12	1	183.33	137.33
Au +N*BP+L*MoS_2_	45	10	1	163.33	126
Au +N*BP+L*WS_2_	53	9	1	183.33	133.333
Au +N*BP+L*MoSe_2_	50	10	1	163.33	129.33
Au +N*BP+L*WSe_2_	54	10	1	180	136

**Table 5:** Optimization results.

Configuration	Silver (nm)	BP (L)	Graphene (L)	MoS_2_ (L)	WS_2_ (L)	MoSe_2_ (L)	WSe_2_ (L)	S_max_	S_1.330__–__1.355_/S_ave_	FOM_1.330__–__1.355_
1	53	12	0	0	0	0	0	380	320	52.46
2	49	12	0	0	0	0	0	400	308	49.68
3	42	12	1	0	0	0	0	320	276	36.32
4	52	9	1	1	0	0	0	260	236	26.52
5	66	7	0	0	1	0	1	300	268	34.81
6	50	7	0	0	1	1	0	280	244	28.37
7	48	5	0	0	2	0	1	280	252	30.00
8	54	1	3	1	1	1	1	200	192	17.14
